# Sildenafil Enhances Neurogenesis and Oligodendrogenesis in Ischemic Brain of Middle-Aged Mouse

**DOI:** 10.1371/journal.pone.0048141

**Published:** 2012-10-31

**Authors:** Rui Lan Zhang, Michael Chopp, Cynthia Roberts, Min Wei, Xinli Wang, Xianshuang Liu, Mei Lu, Zheng Gang Zhang

**Affiliations:** 1 Department of Neurology, Henry Ford Hospital, Detroit, Michigan, United States of America; 2 Physics Department, Oakland University, Rochester, Michigan, United States of America; 3 Department of Biostatistics and Research Epidemiology, Henry Ford Hospital, Detroit, Michigan, United States of America; University of South Florida, United States of America

## Abstract

Adult neural stem cells give rise to neurons, oligodendrocytes and astrocytes. Aging reduces neural stem cells. Using an inducible nestin-CreER^T2^/R26R-yellow fluorescent protein (YFP) mouse, we investigated the effect of Sildenafil, a phosphodiesterase type 5 (PDE5) inhibitor, on nestin lineage neural stem cells and their progeny in the ischemic brain of the middle-aged mouse. We showed that focal cerebral ischemia induced nestin lineage neural stem cells in the subventricular zone (SVZ) of the lateral ventricles and nestin expressing NeuN positive neurons and adenomatous polyposis coli (APC) positive mature oligodendrocytes in the ischemic striatum and corpus callosum in the aged mouse. Treatment of the ischemic middle-aged mouse with Sildenafil increased nestin expressing neural stem cells, mature neurons, and oligodendrocytes by 33, 75, and 30%, respectively, in the ischemic brain. These data indicate that Sildenafil amplifies nestin expressing neural stem cells and their neuronal and oligodendrocyte progeny in the ischemic brain of the middle-aged mouse.

## Introduction

The subventricular zone (SVZ) of the lateral ventricle and the subgranular zone (SGZ) of the dentate gyrus in the hippocampus of the adult rodent brain contain neural stem cells that differentiate into neurons, astrocytes and oligodendrocytes throughout animal life [1], [2], [3]. Generation of neurons and oligodendrocytes is attenuated with aging, which starts at middle age (10 months) and is further reduced at ages beyond 20 months in the rodent [4], [5], [6], [7], [8], [9], [10], [11]. The progressive decline of neural stem cells in the SVZ and the SGZ likely leads to reduction of neurogenesis and oligodendrogenesis in the aged rodent [12]. Loss of neural stem cells is associated with reduced expression of genes for neural stem cell markers including nestin [9]. In addition, aged rats exhibit a decrease in the basal levels of cGMP as a consequence of an increase in phosphodiesterase activity [13].

Stroke induces neurogenesis in aged animals, although basal neurogenesis is attenuated in these animals [9], [14], [15]. Administration of growth factors including fibroblast growth factor and vascular endothelial growth further increases neurogenesis under non-ischemic and ischemic conditions in aged animals [14], [15], [16], [17]. We previously demonstrated that elevation of brain cGMP levels by blocking phosphodiesterase type 5 (PDE5) enzyme activity with PDE5 inhibitors including Sildenafil enhances neurogenesis in aged animals after stroke [10], [11]. However, the effect of PDE5 inhibitors on neural stem cells and their progeny in the ischemic brain of aged mouse has not been investigated. An inducible nestin-CreER^T2^ mouse permits in vivo labeling, tracking, and phenotyping of stem cells and their progeny in the adult SVZ and SGZ [18]. In the present study, using this transgenic mouse, we found that Sildenafil enhanced nestin expressing neuronal and oligodendrocyte progenies in the ischemic brain of middle-aged mice.

## Materials and Methods

All experimental procedures were approved by the Institutional Animal Care and Use Committee of Henry Ford Hospital (IACUC # 0970).

### Animal Model of Middle Cerebral Artery Occlusion (MCAO)

A pair of breeding colony of Nestin-CreER^T2^ mice were kindly provided by Dr. Amelia Eisch (University of Texas Southwestern Medical Center) and R26R-stop-YFP mice, a Cre recombinase reporter strain, were purchased from Jackson Laboratory. Male Nestin-CreER^T2^;*R26R-stop-YFP* mice at age of 14 months with the genotype Nestin-CreER^T2^/+;*R26R-stop-YFP/+* were used in the present study [18]. All Nestin-CreER^T2^;*R26R-stop-YFP* mice used in the present study were YFP heterozygotes. The mice received intraperitoneal injection of tamoxifen (300 mg/kg, Sigma, St. Louis, MO) in sunflower seed oil daily for 5 consecutive days [18], [19]. Four out of 28 mice died within 13 days after tamoxifen injection (1 at 3 days, 2 at 7 days and 1 at 13 days). These mice were not included in the present study. Fourteen days after the last tamoxifen injection, the mice were subjected to permanent right MCAO by inserting a 6-0 nylon filament, as previously described [19], [20].

### Experimental Protocols

**Figure 1:**
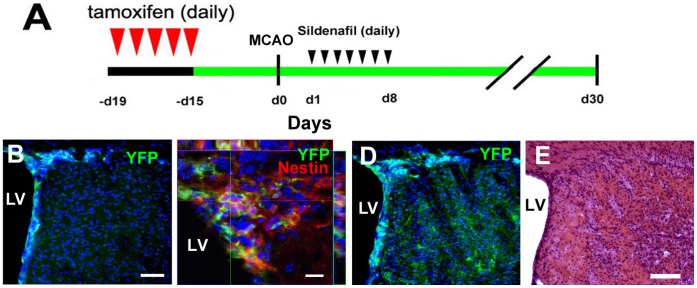
A diagram shows experimental protocols (A). Confocal microscopic images show YFP positive cells in non-ischemic brain 14 days after injection of tamoxifen (B and C) and YFP positive cells in ischemic striatum 30 days after middle cerebral artery occlusion (MCAO, D). An orthogonal view shows that several YFP immunoreactive cells (green) were nestin positive (red) in the anterior SVZ of the lateral ventricle of non-ischemic brain (C). A light microscopic image of hematoxylin and eosin (H&E) stained coronal section shows striatal ischemic lesion 30 days after MCAO (E). Bars = 100 µm for B, and D, 10 µm in C, and 100 µm in E. Blue color  =  cell nuclei. LV  =  lateral ventricle. D0 represents the day of MCAO.

Ischemic mice (n = 12) were treated daily with Sildenafil (subcutaneously) at a dose of 10 mg/kg for 7 consecutive days starting one day after MCAO (Fig. 1A). This dose of Sildenafil has been shown to increase neurogenesis in ischemic brain [10], [21], [22]. Ischemic mice (n = 12) treated with the same volume of saline were used as a control group. The mice were sacrificed 30 days after MCAO.

### Brain Tissue Preparation and Immunohistochemistry

Animals were transcardially perfused with heparinized saline followed by 4% paraformaldehyde. Brains were removed from the skull, fixed further in 4% formaldehyde for 4 h at 4°C and then transferred into 30% sucrose in PBS for 24 h. The brains were embedded and frozen in Optimal Cutting Temperature compound (OCT). A series of 30-µm thick brain coronal sections were cut in a cryostat from bregma 1.18 mm to −0.82 mm for the mouse [23].

Every fifth section was used for immunohistochemistry, as previously described [19], [24]. The following antibodies were used in the present study: mouse anti-nestin (1∶100, BD Bioscience, Franklin, NJ), goat anti-doublecortin (DCX, 1∶200, Santa Cruz Biotechnology, Santa Cruz, CA), mouse anti-NeuN (1∶500, Chemicon/Millipore, Billerica, MA), chicken anti-green fluorescent protein (GFP, 1∶500, Aves Labs, Inc, Tigard, Oregon), rabbit-anti-glial fibrillary acidic protein (GFAP, 1∶10,000, Dako, Carpinteria, CA), rabbit anti-NG2 (1∶800, Chemicon/Millipore), mouse anti-2′,3′-cyclic nucleotide 3′-phosphodiesterase (CNPase, 1∶200, Chemicon/Millipore), and mouse anti-the adenomatous polyposis coli (APC/CC-1, 1∶20, GenWay Biotech. Inc. San Diego, CA). Cell nuclei were stained with 4′, 6′-diamidino-2-phenylindole (DAPI). Double immunofluorescent images were acquired using a Zeiss (Thornwood, NY) LSM 510 Meta NLO system with Coherent Chameleon Ti:Sa laser. Three-color images were scanned using 488 nm argon, 543 HeNe, and Chameleon (750 nm for DAPI) lasers.

### Quantification of YFP^+^ Cells

Stereological unbiased estimates of the total numbers of YFP^+^ cells within the regions of interest were obtained by using a MCID (Microcomputer Imaging Device) stereology software (3D Fractionator, InterFocus Imaging Ltd, Cambridge England) [19]. Briefly, using the automated optical fractionator method, we drew the corpus callosum, striatal, and SVZ areas on coronal sections at a 4× objective. A higher power (a 60× objective, NA 1.4) was then selected, and the system used random systematic sampling to sample 30% of the defined region. When the system moved to the first location within the region of interest, a counting frame was placed over the selected area. We then counted the number of immunostained cells by focusing up and down and marking targets within the counting frame. Data are presented as an estimate of the total number of YFP+ cells in defined regions.

### Statistical Analysis

Data were evaluated for normality. Data transformation would be considered if data were abnormal. Analysis of variance and covariance (ANCOVA) was used to test group difference between saline and Sildenafil. All data are presented as mean ± SE. Statistical significance was set at *p*<0.05.

## Results

### Sildenafil Increases Nestin Lineage Neurons in Ischemic SVZ and Striatum of Middle-aged Mice

Studies in young adult nestin-CreER^T2^/YFP mice demonstrate that Cre recombinase occurs in nestin expressing neural stem cells in the SVZ one day after injection of tamoxifen [18], [25]. To examine whether tamoxifen activates Cre recombinase in middle-aged mice, non-ischemic nestin-CreER^T2^/YFP mice at age of 14 months were treated with tamoxifen for 5 days and their brains were collected 14 days after the last injection of tamoxifen. Immunofluorescent staining of brain coronal sections revealed that YFP^+^ cells were localized to the SVZ and they were nestin+ (Fig. 1B and C). These data indicate activation of Cre recombinase in nestin expressing cells even in middle-aged mice.

**Figure 2:**
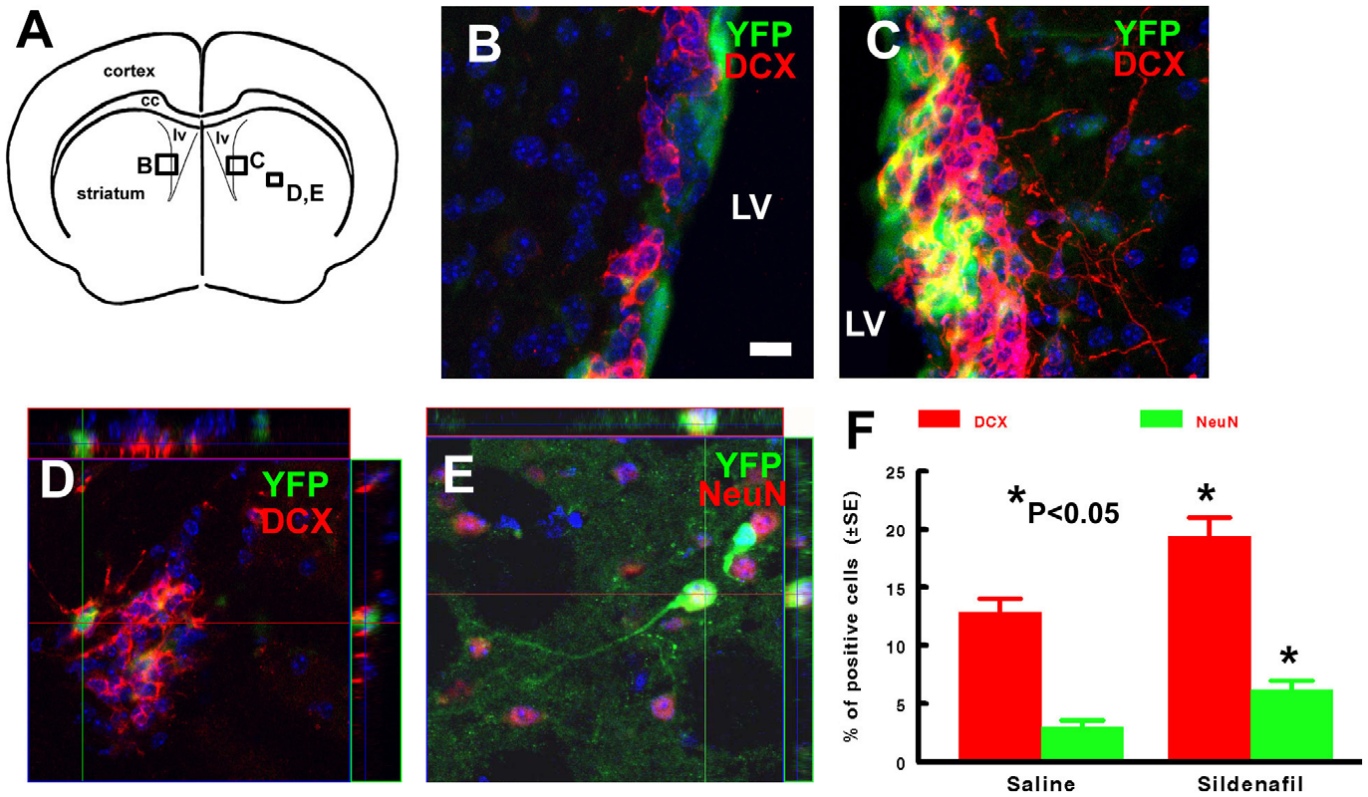
Letters adjacent to boxed areas in schematic representation of a brain coronal section (A) indicate areas where representative confocal microscopic images were taken. Panels B and C show YFP (green) and DCX (red) positive cells in non-ischemic (B) and ischemic SVZ (C) in middle-aged mice 30 days after stroke. Orthogonal views (D, E) show that a YFP immunoreactive cell (green) was DCX positive (D, red) or NeuN (NN) positive (E) in the ischemic striatum. Quantitative data analysis (F) shows percentage of YFP/DCX and YFP/NeuN positive cells in the ischemic striatum after treatment with saline and Sildenafil. *p<0.05 vs the saline group. n = 10/saline and n = 11/Sildenafil. Bar = 10 µm for B to E. Blue color  =  cell nuclei. CC  =  corpus callosum, and LV  =  lateral ventricle.

To examine the effect of stroke on nestin lineage cells in aged mice, the mice were subjected to MCAO two weeks after the last injection of tamoxifen and this time point has been shown for the body clearance of tamoxifen [25]. These mice were sacrificed 30 days after MCAO. Two out of 12 mice died 6 and 21 days after MCAO and these mice were excluded from data analysis. In addition to the SVZ, many YFP+ cells were detected in the striatal ischemic boundary region (Fig. 1C). Unbiased stereology analysis (n = 10 mice) shows that stroke increased the number of YFP+ cells by 30% (2,580±117 vs 1,987±89 cells in the contralateral, p<0.05), 46% (656±41 vs 449±34 cells in the contralateral, p<0.05), and 588% (3,029±114 vs 440±46 cells in the contralateral, p<0.05) in the corpus callosum, SVZ, and striatum of the ipsilateral hemisphere compared to the number in the homologous regions of the contralateral hemisphere, respectively, indicating that stroke increases nestin lineage cells. Phenotype analysis with double immunofluorescent staining revealed that 13% of YFP+ cells (n = 578 cell counted) in the ischemic striatum were DCX+ neuroblasts (Fig. 2B to D, F), while 3% (n = 528 cells counted) of YFP+ cells were NeuN+, a mature neuronal marker, in the ischemic striatum (Fig. 2E, F). These data indicate that stroke increases nestin lineage cells and that nestin lineage cells contribute to generation of new neurons in middle-aged mice.

Our previous studies demonstrated that Sildenafil substantially increased neurogenesis in the ischemic brain of the aged rodent [10], [11]. Aged mice exhibit considerable reduction of nestin expression in SVZ neural stem/progenitor cells [9]. Therefore, we sought to further examine the effect of Sildenafil on the nestin lineage neuronal progeny of middle-aged mice. One mouse died 14 days after MCAO, which was excluded from the data analysis. We found that treatment of stroke with Sildenafil for 7 days (n = 11 mice) substantially increased the number of YFP+ cells in the corpus callosum, SVZ, and striatum of the ipsilateral hemisphere 30 days after MCAO compared to the saline treatment (Table 1). Double immunofluorescent staining showed that Sildenafil treatment increased DCX+ cells from 13% in the control to 19% and NeuN+ cells from 3% to 6% (Fig. 2F). The ischemic lesion was not statistically different between the Sildenafil (49±11% of the contralateral area, n = 11) and saline (50±7% of the contralateral area, n = 10) groups. These data indicate that Sildenafil enhances generation of nestin lineage neurons in the ischemic brain of middle-aged mice.

**Table 1:** The number of YFP^+^ cells in the ischemic hemisphere.

Groups	SVZ	striatum	CC
Ischemia + saline (n = 10)	656±41	3,029±144	2,580±117
Ischemia +Sildenafil (n = 11)	803±28*	3,739±162*	2,958±86*

Date are presented as Mean ± SE. CC  =  corpus callosum. SVZ  =  subventricular zone.

* =  P<0.05 vs the saline group.

### Sildenafil Increases Nestin Lineage OPCs and Mature Oligodendrocytes in the Ischemic Brain of Middle-aged Mice

**Figure 3:**
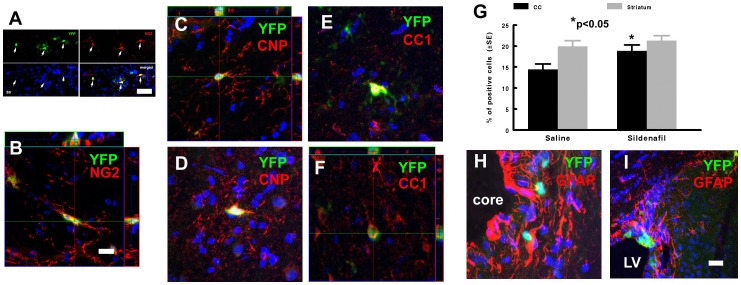
Representative confocal microscopic images (A to F, H, I) show that YFP immunoreactive cells (green) were NG (A, B, red), CNPase (C, D, red), CC1 (E, F, red), and GFAP (H, I, red) positive in the corpus callosum (A, C, E), striatum (B, D, F, H) and SVZ (I) of the ischemic hemisphere in middle-aged mice 30 days after stroke. Panel G shows percentage of YFP/CNPase positive cells in the ipsilateral corpus callosum and striatum in middle-aged mice treated with saline and Sildenafil. *p<0.05 vs saline group. n = 10/saline and n = 11/Sildenafil. Bars  = 10 µm. Blue color  =  cell nuclei. CC  =  corpus callosum, LV  =  lateral ventricle.

In addition to generating new neurons, nestin expressing neural stem cells differentiate into OPCs, mature oligodendrocytes and astrocytes [18], [25], [26], [27], [28]. A study in young adult nestin-CreER^T2^/YFP mice showed that stroke increases nestin lineage OPCs, but not mature oligodendrocytes [25]. We found that YFP+ cells were NG2+ in the ischemic corpus callosum and striatum of middle-aged mice (Fig. 3A, B). Moreover, 15% (673 cells counted) and 18% (614 cell counted) of YFP+ cells in the ischemic corpus callosum and striatum, respectively, were CNPase+, a marker of mature oligodendrocytes (Fig. 3C, D, G). To further confirm that nestin lineage cells are oligodendrocytes, double immunofluorescent staining was performed with another antibody against oligodendrocyte surface marker, CC1 [29]. Confocal microscopy analysis revealed the presence of YFP+ and CC1+ cells in the ischemic corpus callosum and striatum (Fig. 3E, F). These data indicate that nestin linage cells contribute to oligodendrocytes in the ischemic brain of middle-aged mice.

To examine the effect of Sildenafil on nestin-lineage oligodendrocytes in ischemic brain of middle-aged mice, we measured YFP+ and CNPase+ cells in middle-aged nestin-CreER^T2^/YFP mice after stroke. Compared to ischemic animals treated with the saline, ischemic mice treated with Sildenafil exhibited significant increases in the percentage of YFP/CNPase+ cells in the ischemic corpus callosum (702 cells counted), but not the ischemic striatum (757 cells counted) (Fig. 3G), indicating that Sildenafil enhances generation of nestin-lineage oligodendrocytes.

In contrast to nestin-lineage oligodendrocytes, we only detected sporadic YFP+/GFAP+ cells in the striatal ischemic boundary region where activated GFAP+ astrocytes were present (Fig. 3H). However, YFP+/GFAP+ cells were detected in the ipsilateral SVZ 30 days after stroke (Fig. 3I). Sildenafil treatment did not increase the number of YFP+/GFAP+ cells (data are not shown).

## Discussion

The present study shows that stroke induced nestin lineage neuronal and oligodendrocyte progeny and that Sildenafil significantly increased stroke-induced nestin lineage neuronal and oligodendrocyte progeny in middle-aged mice. These data indicate that nestin lineage cells contribute to stroke-induced neurogenesis and oligodendrogenesis in middle-aged mice and that Sildenafil amplifies these processes.

Using the same transgenic mouse line, a previous study in the young adult showed stroke induces nestin expressing neuronal progeny [25]. Our data show that in response to stroke, nestin expressing cells in the SVZ of aged mice differentiated into neurons in the ischemic brain, although aged mice exhibit loss of nestin lineage neural stem cells in the SVZ [9]. We previously demonstrated that Sildenafil augments neurogenesis and improves neurological outcome in aged rats after stroke [10], [11]. The present study confirms and extends our previous work by showing that Sildenafil increases nestin linage neuronal progeny in the ischemic brain. Aging does not appear to affect survival and maturation of neuroblasts [9], [14], [15]. Thus, Sildenafil mediates expansion of nestin lineage neuronal progeny in the ischemic boundary region of middle-aged mice likely by acting primarily on SVZ neural stem cells. These data are consistent with findings by others that aged SVZ neural stem cells retain the ability to respond to injury and exogenous factors similar to adult neural stem cells [9], [14], [15]. Given that aged neural stem cells generate neurons with similar morphology and electrophysiological properties as neurons produced by young adult neural stem cells [9], the nestin lineage neuronal progeny observed in the present study likely have similar function as their counterparts in adult mice.

Aging reduces oligodendrocytes in rodent and human brains [8], [30]. Oligodendrocytes are highly sensitive to ischemic damage [19], [31], [32], [33]. By labeling YFP+ oligodendrocyte progenitor cells and YFP+ neural stem cells in the SVZ of the Nestin-CreER^T2^/+;R26R-stop-YFP/+ mouse prior to induction of stroke, the present study shows that inhibition of PDE5 by Sildenafil increased nestin lineage oligodendrocytes in ischemic boundary regions of the corpus callosum of middle-aged mice. This increase in mature oligodendrocytes is observed by using two antibodies (CNPase and CC1) to intracellular markers that primarily label the cell bodies of mature oligodendrocytes [29], indicating that in addition to neurogenesis, Sildenafil enhances oligodendrogenesis in ischemic brain of middle-aged mice. The finding that Sildenafil expands the population of nestin lineage oligodendrocytes strongly suggests that Sildenafil also acts on oligodendrocyte progenitor cells since mature oligodendrocytes do not proliferate and new oligodendrocytes are derived from non-myelinating oligodendrocyte progenitor cells [34]. The corpus callosum of the adult rodent contains heterogeneous oligodendrocyte progenitor cells [35]. The neural stem cells in the SVZ differentiate into oligodendrocyte progenitor cells that migrate to the ischemic boundary region where they become mature oligodendrocytes [31], [36]. Both populations of nestin lineage oligodendrocyte progenitor cells could contribute to Sildenafil-enhanced oligodendrogenesis in the ischemic brain. Mature oligodendrocytes myelinate axons [37]. Accordingly, increased oligodendrocytes observed in the present study can potentially myelinate axons in the ischemic brain. Moreover, elevation of cGMP by Sildenafil enhances neurogenesis, angiogenesis, and synaptic plasticity, which are closely associated with improvements of neurological outcome and memory in animal models of stroke and Alzheimer’s disease, respectively [10], [21], [38], [39]. Therefore, we speculate that increased neurogenesis and oligodendrogenesis observed in the present study interweave with the other events amplified by Sildenafil, subsequently leading to improved functional outcome after stroke.

We previously demonstrated that adult SVZ neural progenitor cells express PDE5 and that elevation of cGMP levels by Sildenafil substantially augments proliferation and neuronal differentiation of neural progenitor cells [10], [21], [39]. In vitro, Sildenafil or cGMP activates Akt, whereas blockage of the PI3K/Akt signaling pathway with pharmacological inhibitors suppresses Akt activation and subsequently Sildenafil-induced neurogenesis. These data suggest that the PI3K/Akt signaling pathway that regulates adult neurogenesis plays an important role in Sildenafil-induced neurogenesis [39]. In addition to neurogenesis, Sildenafil promotes angiogenesis that couples with neurogenesis and oligodendrogenesis [11], [40], [41], [42], [43], [44]. Thus, Sildenafil could amplify neurogenesis and oligodendrogenesis by directly acting on neural progenitor and oligodendrocyte progenitor cells, by acting indirectly through enhanced angiogenesis, or by both. Future studies of individual contributions are warranted.

The present study showed the presence of YFP+/GFAP+ cells in the SVZ 44 days after tamoxifen injection, which is consistent with the fact that a subpopulation of astrocytes in the SVZ are neural stem cells that express nestin [2], [18]. Under non-ischemic condition, Lagace et at demonstrate that nestin expressing neural stem cells (YFP+/GFAP+/SOX2+ cells) are present in the SVZ and they do not give rise to astrocytes in the same inducible nestin-CreER^T2^ mouse line [18]. Interestingly, we only observed few YFP+/GFAP+ cells in ischemic boundary regions in the middle-age mouse. However, a previous study in the young adult mouse showed that 59% of YFP+ cells were GFAP+ in the ischemic boundary regions [25]. We do not know the causes for this discrepancy. One major difference between our study and Li et al one is animal age, i.e., 14 months vs 8 weeks in Li et al study. Animal age could affect induction of Cre recombinase activity [45], which may contribute to sub-optimal detection of YFP signals. Moreover, the current study analyzed YFP expressing cells only at one time point (30 days after stroke), whereas Li et al measured nestin expressing cells at multiple time points after stroke. Furthermore, the present study did not examine the fate of other 48% of YFP positive cells, although some of YFP positive cells exhibited oligodendrocyte progenitor cell phenotype. We speculate that these YFP positive cells could remain in neural progenitor stage and have potential to differentiate into glial cells or neurons.

In summary, the present study demonstrates that Sildenafil enhances not only nestin lineage neurogenesis, but also oligodendrogenesis in the ischemic brain of the middle-aged mouse, which provides new insight into the therapeutic effect of Sildenafil on brain repair after stroke.
